# Particle-level residence time data in a twin-screw feeder

**DOI:** 10.1016/j.dib.2019.104672

**Published:** 2019-10-18

**Authors:** Peter Toson, Johannes G. Khinast

**Affiliations:** a Research Center Pharmaceutical Engineering (RCPE), Inffeldgasse 13, 8010, Graz, Austria; b Institute of Particle and Process Engineering (IPPT), Inffeldgasse 13, 8010, Graz, Austria

**Keywords:** Discrete element method, Twin-screw feeder, Residence time distribution, Pharmaceutical engineering

## Abstract

A full discharge process of a twin-screw feeder has been simulated with DEM (discrete element method). The result files are available at the Mendeley Data repository (https://doi.org/10.17632/d76rzzd8r7.1) and contain the following particle data: x,y,z coordinates of the initial position inside the feeder, particle radius, and the discharge time of each particle are available at three different initial feeder fill levels. With this data it is possible to generate residence time distributions (RTDs) of arbitrary spatial regions in the feeder to analyze the material flow inside the feeder, optimize refill strategies, and ultimately improve batch definition in continuous manufacturing. Example RTDs and evaluation scripts are available in the repository.

**Table:** Specifications Table

Subject area	Chemical Engineering
More specific subject area	Pharmaceutical Engineering, Powder Processing
Type of data	particle-based data for 3 feeder fill levels (3 text files), 24 example cumulative distributions (3 text files), example script to generate RTDs (1 python script), video of the full discharge process rendered from raw DEM results (1 avi file with mpeg4 encoding)
How data was acquired	DEM (discrete element method) simulations
Data format	raw and analyzed data, analysis script
Experimental factors	sampling time for checking particle discharge times: every 0.02s
Experimental features	DEM software package: XPS + python scripting
Data source location	Graz, Austria: Research Center Pharmaceutical Engineering (47.0593 N,15.4633 E)
Data accessibility	Mendeley Data. https://doi.org/10.17632/d76rzzd8r7.1

**Table:** 

**Value of the Data** • This dataset contains residence times of individual particles in a twin-screw feeder obtained from DEM (discrete element method) simulations. With this dataset it is possible to obtain residence time distributions (RTDs) to characterize the discharge process. • The residence time data in this dataset can be used to model material tracking in a continuous pharmaceutical production process through RTD modeling [1]. • Obtaining the same or similar data with experiments is difficult: Experimental determination of RTDs in feeders is material intensive and requires one experiment for each examined fill level [2]. The data can be used to find an optimal fill level and refill strategy, and designing an experiment for confirmation. • Starting positions of particles inside the feeder are included in the dataset, which allows the definition and analysis of arbitrary spatial sub-regions of the feeder. The RTDs included in this article are and can only be exemplary. An example script to analyze the RTDs in two regions is included in the dataset.

## 1 Data

**Fig. 1:**
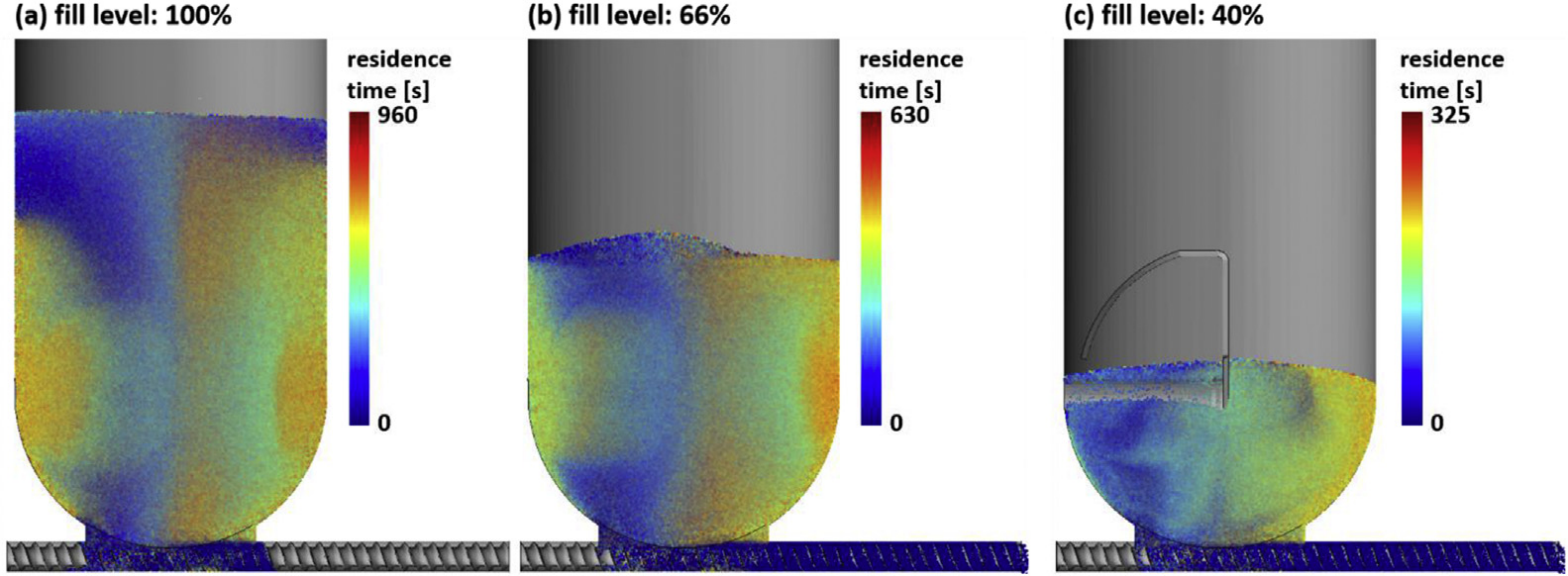
Graphical representation of the data in the discharge-times_XXX.txt files. Particles are rendered semi-transparent. The feeder geometry is not part of the dataset but is shown for clarity.

**Fig. 2:**
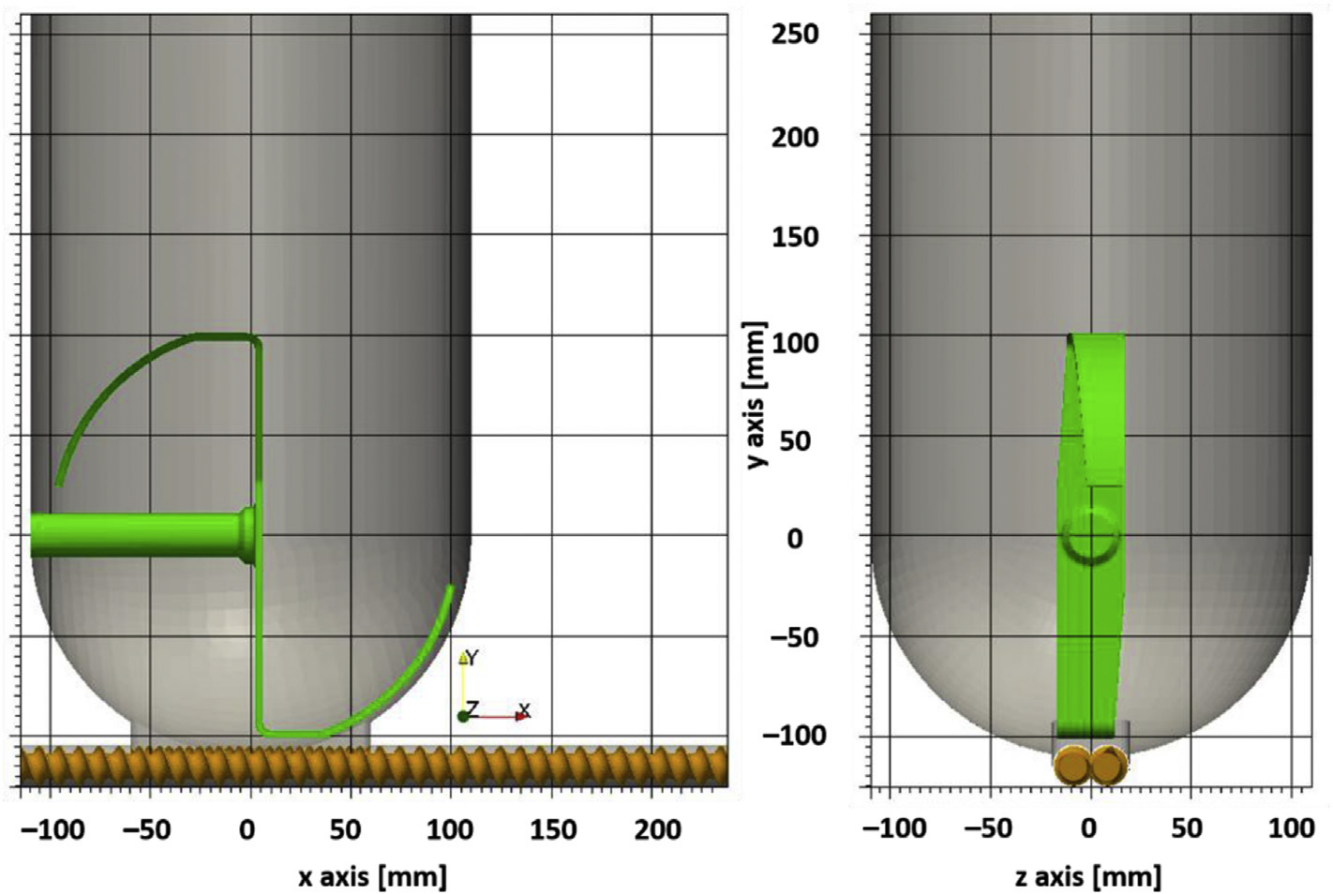
Dimensions and coordinate system of the feeder in the DEM simulation.

**Fig. 3:**
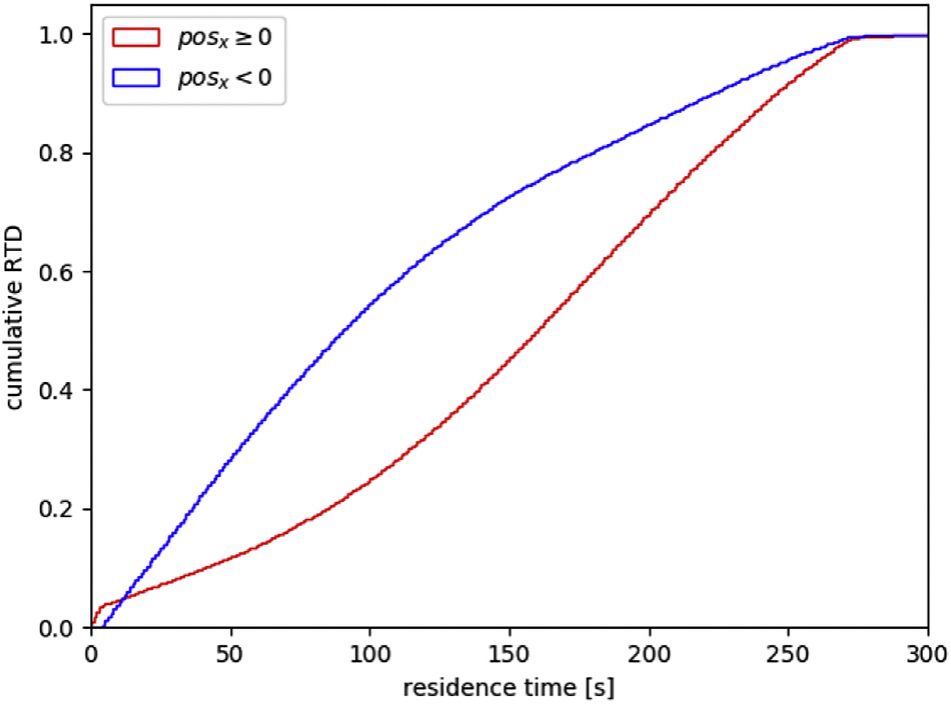
Result of the script minimalworkingexample.py.

**Fig. 4:**
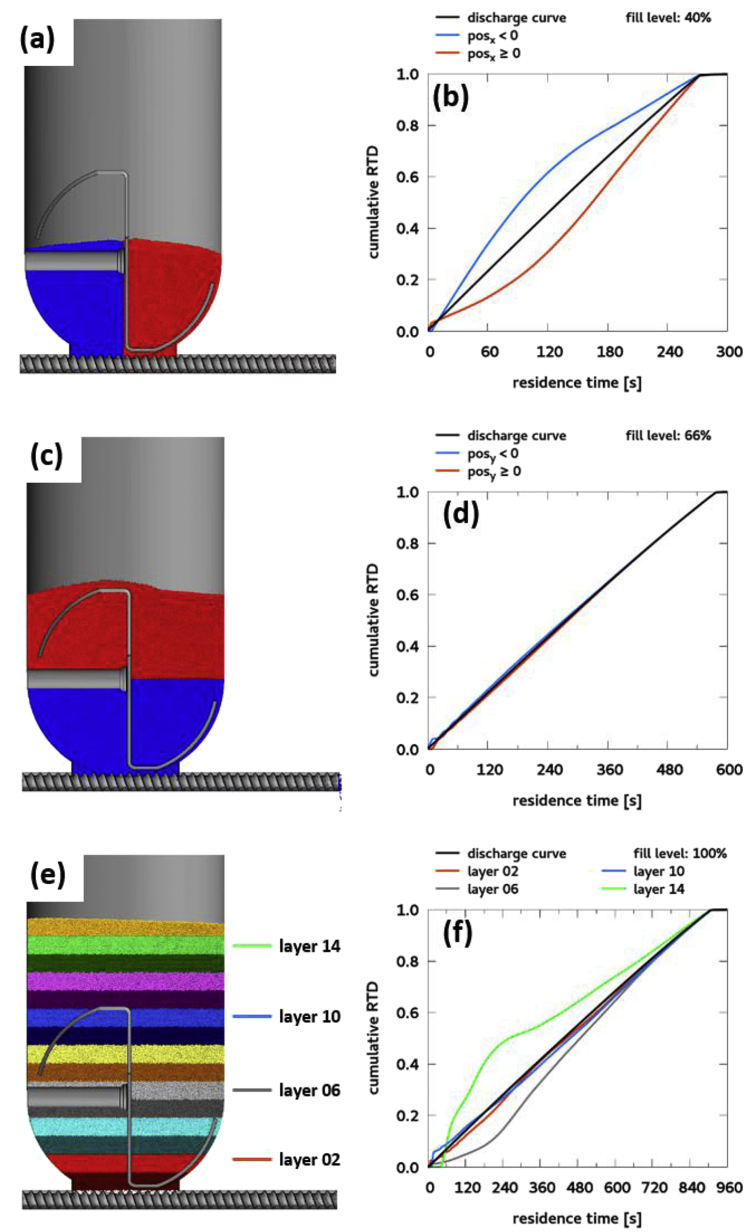
Example regions and cumulative residence time distributions obtained from the dataset. (a) 40% fill level, region defined by x coordinate. (b) Corresponding RTD curve. (c) 66% fill level, region defined by y coordinate. (d) Corresponding RTD curve. (e) 100% fill level, regions are 2cm thick slices of the particle bed. (f) Example RTD curves.

The data is based on DEM simulations of a feeder discharge process. The video *kt20_discharge_08Mbits.avi* is a rendered from raw DEM data and shows the discharge from 100% fill level to empty in 16 minutes. The text files *discharge-times_040.txt*, *discharge-times_066.txt*, and *discharge-times_100.txt* contain the data for one particle per line and have the following columns: starting position of the particles (x, y, z in meters), residence time of the particle in seconds, and the particle radius in meters. The number in the file name corresponds to the initial feeder fill level: 40%, 66%, 100%. Fig. 1 shows the residence times of particles at different fill levels, Fig. 2 shows the feeder geometry and coordinate system used in the simulation. The python script *minimalworkingexample.py* analyses the data at 40% fill level and plots the cumulative RTDs of two regions in the feeder that are defined by the sign of the x coordinate (Fig. 3). The dataset contains the following example cumulative distributions:

• *kt20_cumulative_040_x.txt*: 40% fill level, regions are defined by the sign of the x coordinate (Fig. 4a, b)
• *kt20_cumulative_066_y.txt*: 66% fill level, regions are defined by the sign of the y coordinate (Fig. 4c, d)
• *kt20_cumulative_100_layers.txt*: 100% fill level, regions are 2cm thick layers of powder defined by the y coordinate (Fig. 4e). RTD data is available for all 16 layers (layer 0 corresponds to particles initially in the screw), data for four layers are shown in Fig. 4f.

## 2 Experimental design, materials, and methods

The DEM data has been generated with the software package XPS (extended particle system). XPS is a high-performance GPU-based code and has been successfully applied to a wide range of industry-scale applications in the pharmaceutical field, e.g. tablet coating [3], batch and continuous mixing [4,5], and fluidized bed coating [6]. Implementation details are given in Refs. [5,7].

An STL model of a KTron KT20 twin-screw feeder has been created and imported to XPS (Fig. 2). The feeder model contains twin concave screws with a pitch of 2cm. The agitator and screw speeds have been held constant during the simulation (volumetric feeding).The DEM simulations used the linear spring dashpot contact model without any cohesive forces. The contact model, simulation, and process parameters are shown in Table 1. The simulation contained 2.5 M particles and ran at an average of 36 integration time steps per second on a single GPU (Nvidia GTX 1080Ti). The discharge process took 960 process seconds and the simulation finished within 2 months. Every 0.02 process seconds, a complete DEM snapshot containing particle position, velocity, contact and geometry information has been written. One snapshot is has a file size of 150MB. The complete DEM raw data has a total size of 2.7TB and is not part of the dataset.

**Table 1:** Contact model, simulation, and process parameters.

Contact stiffness k	2000 N/m
particle-particle sliding friction $\mu_{PP}$	0.5
particle-wall sliding friction $\mu_{PW}$	0.5
particle rolling friction $\mu_{r}$	0.1
normal and tangential restitution coefficient $e_{n},\;e_{t}$	0.5
particle diameter: mean and standard deviation	800 ± 600 μm
particle diameter: min and max	550–1100 μm
DEM time step Δt	5 μs
number of particles	2,500,000
agitator speed	36 rpm
screw speed	180 rpm
process time	960 s

The particle residence times in the dataset are generated in post-processing by analyzing the written DEM snapshots. The residence time for each particle is defined as the time between the start of the evaluation and the first time step where the particle is outside of the bounding box indicated in Fig. 2. The start of evaluation for the 100% fill level data is t_0_ = 0s, the data for lower fill levels are generated by starting the analysis at a later time step (t_0_ = 330s for 66%, t_0_ = 635s for 40% fill level). The screws are already filled at the lower fill levels, whereas they are empty in the 100% fill level analysis. The RTDs are then generated by histogramming the residence times of the individual particles to determine the refill behavior (Fig. 4c, d) and to analyze the particle flow inside the feeder (simple examples in Fig. 3 and Fig. 4a, b, complex example in Fig. 4e, f).
